# Interactions of Protonated Guanidine and Guanidine Derivatives with Multiply Deprotonated RNA Probed by Electrospray Ionization and Collisionally Activated Dissociation

**DOI:** 10.1002/open.201700143

**Published:** 2017-10-24

**Authors:** Jovana Vušurović, Eva‐Maria Schneeberger, Kathrin Breuker

**Affiliations:** ^1^ Institute of Organic Chemistry and Center for Molecular Biosciences Innsbruck (CMBI) University of Innsbruck Innrain 80–82 6020 Innsbruck Austria

**Keywords:** collisionally activated dissociation, electrospray ionization, guanidinium, mass spectrometry, RNA

## Abstract

Interactions of ribonucleic acid (RNA) with guanidine and guanidine derivatives are important features in RNA–protein and RNA–drug binding. Here we have investigated noncovalently bound complexes of an 8‐nucleotide RNA and six different ligands, all of which have a guanidinium moiety, by using electrospray ionization (ESI) and collisionally activated dissociation (CAD) mass spectrometry (MS). The order of complex stability correlated almost linearly with the number of ligand atoms that can potentially be involved in hydrogen‐bond or salt‐bridge interactions with the RNA, but not with the proton affinity of the ligands. However, ligand dissociation of the complex ions in CAD was generally accompanied by proton transfer from ligand to RNA, which indicated conversion of salt‐bridge into hydrogen‐bond interactions. The relative stabilities and dissociation pathways of [RNA+*m* L−*n* H]^*n*−^ complexes with different stoichiometries (*m*=1–5) and net charge (*n*= 2–5) revealed both specific and unspecific ligand binding to the RNA.

## 1 Introduction

Interactions of ribonucleic acids (RNA) with proteins and small molecules play an important role in many fundamental biological processes.^1^ RNA–protein^2^ and RNA–drug^3^ complex interfaces are often stabilized by stacking, cation–π, hydrogen‐bond, and salt‐bridge interactions2b, 4 between guanidinium functionalities and RNA. For example, arginine residues are frequently found in the RNA‐binding regions of proteins,2b, 5 such as those in the human ribosomal protein L7 and the human immunodeficiency virus type 1 (HIV‐1) rev protein,^6^ and many pharmaceutically active compounds contain guanidinium moieties,^7^ such as antihypertensive drugs (e.g., amiloride, clonidine, guanethidine), antidiabetics (e.g., metformin, buformin, galegin), and antibiotics (e.g., streptomycin, sulfaguanidine).^8^

RNA–ligand complexes can be studied by using computational^9^ or experimental approaches such as nuclear magnetic resonance (NMR) spectroscopy,3a, 10 X‐ray crystallography,^11^ biochemical methods,^12^ and crosslinking strategies.12a, 13 Although highly promising native mass spectrometry (MS) studies of RNA–protein^14^ and RNA–drug^15^ complexes have continued to appear in the literature over the past 20 years, they are still scarce compared with those for protein–protein interactions.^16^ This is quite surprising in view of the high stability of guanidinium–phosphate interactions in gaseous ions^17^ and the inherent advantages of native MS, for example, that it does not require stable isotope labeling or crystallization, is not limited by crosslinking reactivity, and uses only relatively small quantities of sample material compared with NMR spectroscopy and X‐ray crystallography. Moreover, a number of laboratories have reported that in the gas phase, the strength of noncovalent bonds between RNA14g, 15e, 17b, 18 or deoxyribonucleic acid (DNA)17d and basic ligands can even exceed those of covalent bonds. As a case in point, we have recently shown that the noncovalent bonds between trans‐activation responsive (TAR) RNA and a peptide with the arginine‐rich binding region of the trans‐activator of transcription (tat) protein from HIV‐1 are sufficiently strong to survive phosphodiester backbone cleavage of the RNA by collisionally activated dissociation (CAD), which thus allowed its use to probe tat binding sites of TAR RNA by top‐down MS.14g

The unusual stability of noncovalent bonds in the gas phase has been attributed to strong electrostatic interactions,14g such as salt bridges, ionic and neutral hydrogen bonds, and charge–dipole interactions,^19^ of which salt bridges were thought to provide the highest contribution to stability.^20^ In support of this hypothesis, calculations suggest that the interaction energy between guanidine and trifluoroacetic acid, that is, the stabilization achieved when the two neutral molecules are brought from infinite distance to equilibrium distance, is ≈70 kJ mol^−1^, whereas that of protonated guanidine and trifluoroacetate, that is, the stabilization achieved when the two oppositely charged ions are brought from infinite distance to equilibrium distance, is ≈500 kJ mol^−1^.^21^ These energies differ by almost an order of magnitude, which highlights the fact that the balance between covalent and noncovalent bond dissociation critically depends not only on the number but also the type of interactions.

Here, we used electrospray ionization (ESI) and low‐energy CAD to systematically study the binding of basic ligands to an 8‐nucleotide (8‐nt) RNA. All ligands investigated (Table 1), that is, guanidine (Gnd), 1‐methylguanidine (meGnd), 1,1,3,3‐tetramethylguanidine (tmeGnd), 3‐guanidinopropanoic acid (Gpa), l‐2‐amino‐3‐guanidinopropanoic acid (aGpa), and l‐arginine (Arg), contain (substituted) guanidinium moieties with p*K*_a_ values between 12.6 and 13.8,^22^ and vary both in proton affinity (PA) and the types and number of intra‐ and intermolecular interactions that they can form. As a basis for the further development of top‐down native MS for the detection of RNA–protein complexes and the characterization of their binding interfaces, we address the relative strengths of individual interactions, the competition between noncovalent and covalent bond cleavage, binding specificity, the energetics of intermolecular proton transfer, and the effect of the complex net charge.

**Table 1:** Chemical structures of the ligands studied.

Ligand (L)	Chemical Structure
guanidine (Gnd)	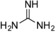
1‐methylguanidine (meGnd)	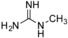
1,1,3,3‐tetramethylguanidine (tmeGnd)	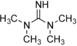
3‐guanidinopropanoic acid (Gpa)	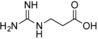
l‐2‐amino‐3‐guanidinopropanoic acid (aGpa)	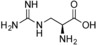
l‐arginine (Arg)	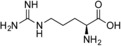

## 2 Results and Discussion

### 2.1 ESI MS of RNA–Ligand Complexes

**Figure 1:**
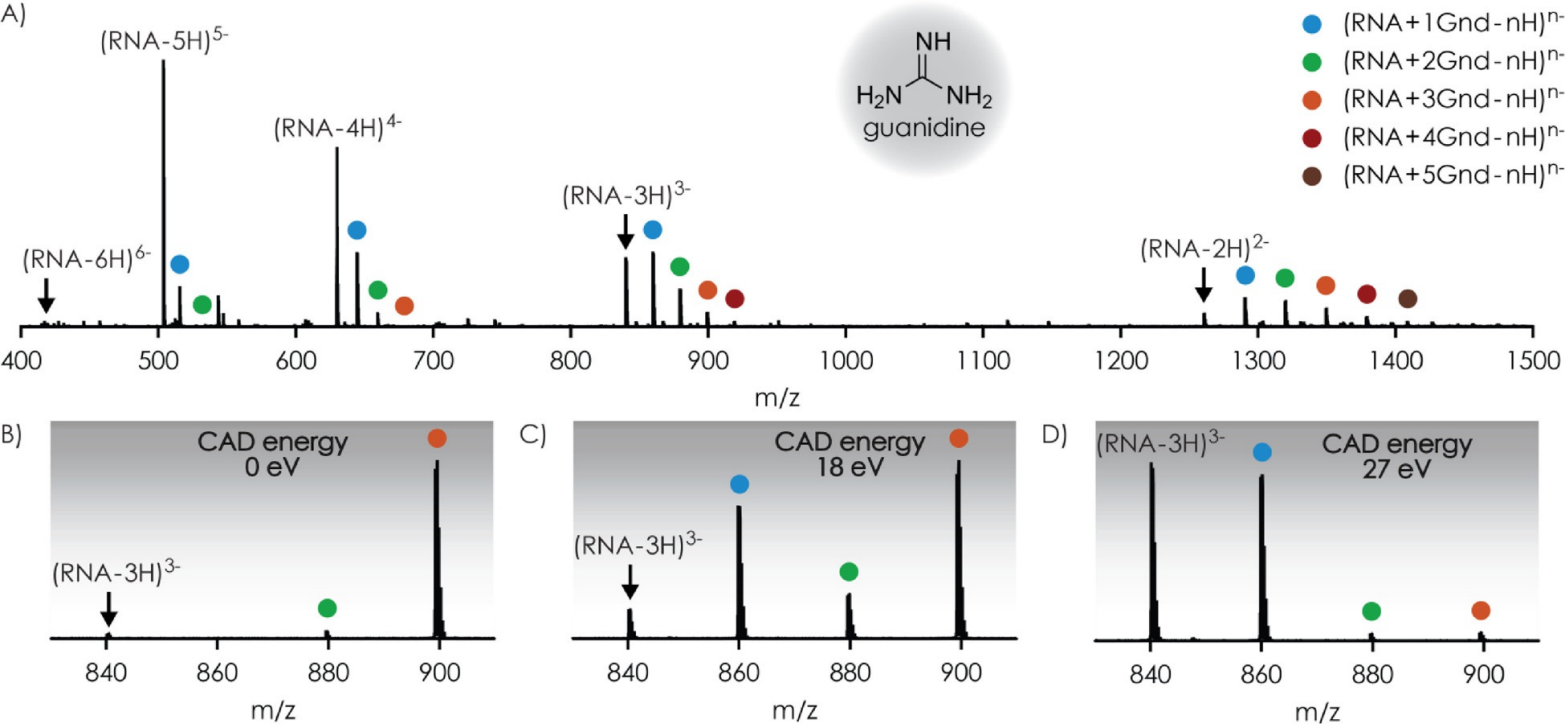
Mass spectra from A) ESI of a 1 μm RNA solution in 1:1 H_2_O/CH_3_OH with 100 μm guanidine at pH≈7.5, B) isolation of [RNA+3 Gnd−*n* H]^*n*−^ ions, and subsequent CAD at C) 18 and D) 27 eV laboratory‐frame energy; colored circles indicate the number of Gnd ligands bound to the 8‐nt RNA.

Noncovalently bound complexes of the 8–nt RNA (GGCUAGCC, 5′‐OH and 3′‐OH termini) and guanidine or guanidine derivatives (Table 1) were produced by electrospraying solutions of 1 μm RNA and 5–100 μm ligand (L) in 1:1 H_2_O/CH_3_OH at pH ≈7.5, adjusted by the addition of piperidine and imidazole (≈1 mm each). The additive mixture of piperidine and imidazole was used because it very efficiently suppresses the formation of sodium and potassium adducts without promoting formation of highly charged ions.^23^ 8‐nt RNA contains all canonical nucleobases and, according to theoretical predictions (http://rna.tbi.univie.ac.at/cgi‐bin/RNAWebSuite/RNAfold.cgi),^24^ should not form any stable secondary structures in solution. However, the RNA sequence is self‐complementary and a high methanol content along with a low RNA concentration was used to prevent dimer formation;^25^ dimer ions were not observed in any of the ESI spectra recorded in this study. The near‐neutral pH of ≈7.5, at which the guanidine moiety of all ligands should be protonated (Table 1) and the RNA phosphodiester moieties deprotonated,^26^ was chosen to promote the formation of intermolecular salt bridges between the ligand guanidinium and RNA phosphodiester moieties in solution. Under these conditions, RNA–ligand complex ions, [RNA+*m* L−*n* H]^*n*−^, from ESI were observed for all ligands studied, as illustrated for guanidine in Figure 1A. The net charge *n* on the RNA–ligand complexes ranged from 2 to 5 (Figure 1 and Figure S1 in the Supporting Information), and the proportion of [RNA+*m* L−*n* H]^*n*−^ ions generally increased as *n* decreased, that is, 0 % for *n*=6 and >70 % for *n*=2 (Table S1), which is consistent with each protonated ligand compensating one of the negative charges of the RNA in the association reaction in solution [Reaction (I)]:

(I) $$\left[\mathrm{RNA}-k\;H\right]{}^{k-}+m\;\left[L+H\right]{}^{+}\to \left[\mathrm{RNA}+m\;L-n\;H\right]{}^{n-};\;n=k-m$$

In agreement with previous studies of guanidinium derivatives binding to DNA,^27^ the maximum number of bound ligands (Table 1) did not exceed seven, the number of phosphodiester moieties in the 8‐nt RNA, at the highest ligand concentration used (100 μm; Figure S1), except for Gpa. At this concentration, RNA complex ions with up to 14 Gpa ligands were detected, along with abundant Gpa cluster ions. The latter were not observed for Gnd, meGnd, and tmeGnd, and were found in much lower abundance for aGpa and Arg at a concentration of 100 μm than for Gpa at 10 μm (Figure S1). A possible rationale for the different behavior of Gpa regarding cluster formation and binding to RNA is its net charge, which should be zero assuming that the guanidine and carboxylic acid groups are protonated and deprotonated, respectively, at the solution pH of ≈7.5 used. By contrast, Gnd, meGnd, tmeGnd, aGpa, and Arg should each carry a net charge of +1 at this pH assuming that both the guanidine and amino groups are protonated and the carboxylic acid moieties are deprotonated. In this case, Coulombic repulsion limits cluster formation and binding to RNA for all ligands studied, except for the overall neutral Gpa. Although the RNA complex and ligand cluster ions found in the ESI spectra do not necessarily reflect the species present in solution, these data suggest that the [RNA+*m* L−*n* H]^*n*−^ ions predominantly originate from association reactions in solution.

### 2.2 CAD MS of RNA–Ligand Complexes

To investigate ligand binding to RNA in the gas phase, we isolated [RNA+*m* L−*n* H]^*n*−^ complex ions with different numbers of ligand (*m*) and net charge (*n*) in the mass spectrometer prior to CAD (Figure 1B) at laboratory‐frame energies of up to 75 eV. The dissociation reactions observed here involved sequential loss of neutral ligands [Reaction (II)]:

(II) $$\left[\mathrm{RNA}+m\;L-n\;H\right]{}^{n-}\to \left[\mathrm{RNA}+\left(m-1\right)\;L-n\;H\right]{}^{n-}+L\left[\mathrm{RNA}+\left(m-1\right)\;L-n\;H\right]{}^{n-}\to \left[\mathrm{RNA}+\left(m-2\right)\;L-n\;H\right]{}^{n-}+L\left[\mathrm{RNA}+\left(m-2\right)\;L-n\;H\right]{}^{n-}\to \left[\mathrm{RNA}+\left(m-3\right)\;L-n\;H\right]{}^{n-}+L,\;\mathrm{etc}.$$

and loss of up to two negatively charged ligands [Reaction (III)]:

(III) $$\left[\mathrm{RNA}+m\;L-n\;H\right]{}^{n-}\to \left[\mathrm{RNA}+\left(m-1\right)\;L-\left(n-1\right)\;H\right]{}^{(n-1)-}+\left[L-H\right]{}^{-}\left[\mathrm{RNA}+\left(m-1\right)\;L-\left(n-1\right)\;H\right]{}^{(n-1)-}\to \;\;\;\;\left[\mathrm{RNA}+\left(m-2\right)\;L-\left(n-2\right)\;H\right]{}^{(n-2)-}+\left[L-H\right]{}^{-}$$

**Scheme 1:**
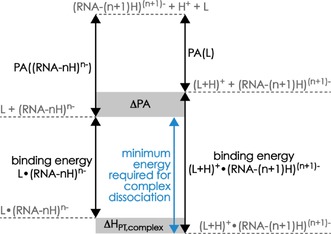
Schematic diagram of the energies associated with ligand dissociation from [RNA+(*m*−1) L−(*n*+1) H]^(*n*+1)−^ **⋅[**L+H]^+^ ions for *m*=1, similar to that for ion pairs in Ref. 21; the minimum energy required for complex dissociation is indicated in blue.

Products from Reaction (IV):

(IV) $$\left[\mathrm{RNA}+m\;L-n\;H\right]{}^{n-}\to \;\;\;\;\left[\mathrm{RNA}+\left(m-1\right)\;L-\left(n+1\right)\;H\right]{}^{(n+1)-}+\left[L+H\right]{}^{+}$$

were not observed in any of the CAD experiments performed herein, which indicates that proton transfer (PT) from protonated ligand to deprotonated RNA had occurred in the gaseous complex ions before ligand dissociation. The energy required for conversion of a salt‐bridge binding motif in [RNA+(*m*−1) L−(*n*+1) H]^(*n*+1)−^ **⋅[**L+H]^+^ complexes into a hydrogen‐bond motif in [RNA+(*m*−1) L−*n* H]^*n*−^ **⋅**L complexes by PT (Δ*H*_PT,complex_) depends on the PA values of the [RNA+(*m*−1) L−(*n*+1) H]^(*n*+1)−^ ions and the neutral ligand L, and the RNA–ligand binding energies of the two complex structures, [RNA+(*m*−1) L−(*n*+1) H]^(*n*+1)−^ **⋅[**L+H]^+^ and [RNA+(*m*−1) L−*n* H]^*n*−^ **⋅**L (Scheme 1).^21^

The ligand PA values are 986, 1032, and 1051 kJ mol^−1^ for Gnd, tmeGnd, and Arg, respectively^28^ (experimental PA values for meGnd, Gpa, and aGpa have not been reported, but calculations^29^ suggest that they also lie in this range), and that of dimethyl phosphate, (CH_3_O)_2_PO_2_^−^, as a model for the deprotonated phosphodiester moiety, is 1387 kJ mol^−1^.^30^ However, the PA values of the deprotonated phosphodiester moieties in RNA can be different from that of dimethyl phosphate as a result of internal hydrogen bonding and charge delocalization.^31^ For example, a PA value of 1279 kJ mol^−1^ was derived in bracketing‐type experiments for adenosine monophosphate,^32^ the gas‐phase structure of which features ionic hydrogen bonding between the phosphate and the 3′‐hydroxyl group, which in turn is hydrogen bonded to the 2′‐hydroxyl group;^33^ that of phosphate is 1383 kJ mol^−1^.^30^

From the above PA values, PT from protonated ligand to dimethyl phosphate, that is, [L+H]^+^+(CH_3_O)_2_PO_2_^−^→L+(CH_3_O)_2_PO_2_H, or to adenosine monophosphate in transiently formed, unstable complexes is highly exothermic (ΔPA, Scheme 1) by 336 to 401 or 228 to 293 kJ mol^−1^, respectively. By contrast, PT from protonated ligand to deprotonated RNA within stable RNA–ligand complexes is an endothermic reaction (Δ*H*_PT,complex_>0 kJ mol^−1^) that requires an energy input to proceed because the binding energies of [RNA+(*m*−1) L− (*n*+1) H]^(*n*+1)−^ **⋅[**L+H]^+^ ions are generally far higher than those of [RNA+(*m*−1) L−*n* H]^*n*−^ **⋅**L ions, as a result of the far higher electrostatic interaction energies of the former.

Therefore, the energy provided by slow ion heating in CAD causes PT within the [RNA+(*m*−1) L−(*n*+1) H]^(*n*+1)−^ **⋅[**L+H]^+^ ions to produce [RNA+(*m*−1) L−*n* H]^*n*−^ **⋅**L ions that can further dissociate into [RNA+(*m*−1) L−*n* H]^*n*−^ and L [Reaction (II), Scheme 1] unless the interconversion barrier between salt‐bridge binding motifs (protonated ligand and deprotonated phosphodiester moiety) and hydrogen‐bond motifs (both ligand and phosphodiester moiety uncharged) is sufficiently high to prevent PT on the timescale of the experiment. Calculated interconversion barriers are far smaller than PT reaction exothermicities;^21^ up to about 18 kJ mol^−1^ for protonated dimers of betaine and ammonia^34^ and 15 to 30 kJ mol^−1^ for overall neutral dimers of guanidine and formic acid.^35^ Although PT barriers in the larger structures studied here likely differ from the above values,^36^ the lack of products from Reaction (IV) suggests that the barriers for interconversion between salt‐bridge and neutral‐binding motifs in the RNA–ligand complexes are too small to prevent PT from protonated ligand to deprotonated RNA.

The branching ratio of products from loss of [L−H]^−^ by Reaction (III) versus loss of neutral ligand L by Reaction (II) was affected by the complex ion net charge *n*, the number of ligands *m* bound to the RNA, the ligand identity, and the energy available for dissociation. For *n*=2 to 3 and all ligands studied, the only products from CAD of [RNA+*m* L−*n* H]^*n*−^ ions were from successive losses of neutral ligand [Reaction (II)]. Moreover, no deprotonated ligand, [L−H]^−^, was detected for tmeGnd, meGnd, or Gnd irrespective of the net charge *n* and the CAD energy used, which suggests that the PAs of [tmeGnd−H]^−^, [meGnd−H]^−^, and [Gnd−H]^−^ far exceed those of the [RNA+(*m*−1) L−*n* H]^*n*−^ ions; a correspondingly high p*K*_a_ value of 28.5 was reported for Gnd.^37^ However, up to 1 % [RNA+(*m*−1) L−(*n*−1) H]^(*n*−1)−^ ions were detected for tmeGnd, meGnd, and Gnd at *n*=4, which can be attributed to PT from evaporated solvent to [RNA+*m* L−*n* H]^*n*−^ ions during the 1 s ion accumulation time in the collision cell. Likewise, CAD of [RNA−4 H]^4−^ and [RNA+L−4 H]^4−^ ions of tmeGnd, meGnd, and Gnd showed <1 % [RNA−3 H]^3−^ ions irrespective of the energy used. PT to [RNA−5 H]^5−^ ions during the 1 s accumulation period was even higher at up to 30 %, whereas no PT was observed for *n*=2 and 3. These data indicate an increasing proton affinity of the [RNA−*n* H]^*n*−^ ions with increasing *n*, similar to the increasing PA of peptide and protein [*M*+*n* H]^*n*+^ ions with decreasing *n*.^38^

Consistent with the PAs of ligand anions comprising carboxylates, for example, ≈1385 kJ mol^−1^ for [Arg−H]^−^,^30^ that are comparable to that of the deprotonated phosphodiester moiety, CAD of [RNA+L−*n* H]^*n*−^ ions with Gpa, aGpa, and Arg for *n*=4 to 5 did produce [L−H]^−^ ions, but because our FT‐ICR instrument relies on charge detection, the [L−H]^−^ ions were detected with a sensitivity up to four times lower than the corresponding multiply charged RNA (complex) ions with *n*=3 and 4. Moreover, time‐of‐flight differences in the transfer of ions with low and high *m*/*z* values (≈58 to ≈173 for [L−H]^−^ versus ≈630 to ≈1261 for the corresponding RNA or RNA complex ions) from the collision to the ICR cells complicate quantitative detection of complementary ionic products from Reaction (III). We thus used only the signals of RNA (complex) ions for further data analysis.

**Figure 2:**
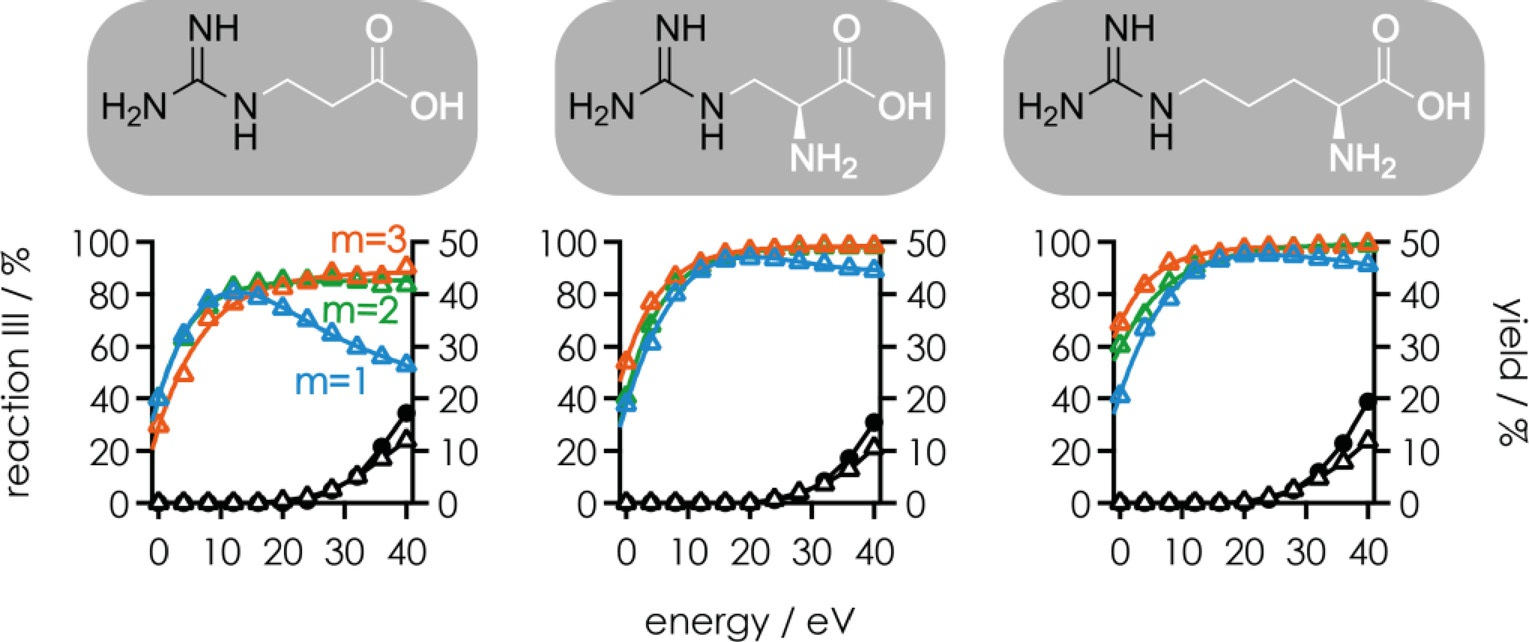
Fraction of products from Reaction (III) out of all products from Reactions (II) and (III) (left axes) in CAD of [RNA+*m* L−4 H]^4−^ ions for *m*=1 (blue), *m*=2 (green), and *m*=3 (orange) for Gpa, aGpa, and Arg (corresponding ligand structures with the guanidine moieties highlighted in black are shown on top) versus laboratory‐frame energy. Also shown are yields (right axes) of ***c***, ***y*** (up to 13.5 %) and ***a***, ***w*** (up to 3.5 %) fragments including those that showed nucleobase loss (black circles), and products of nucleobase loss from [RNA+L−4 H]^4−^ ions (black triangles) in CAD of [RNA+L−4 H]^4−^ ions.

**Scheme 2:**
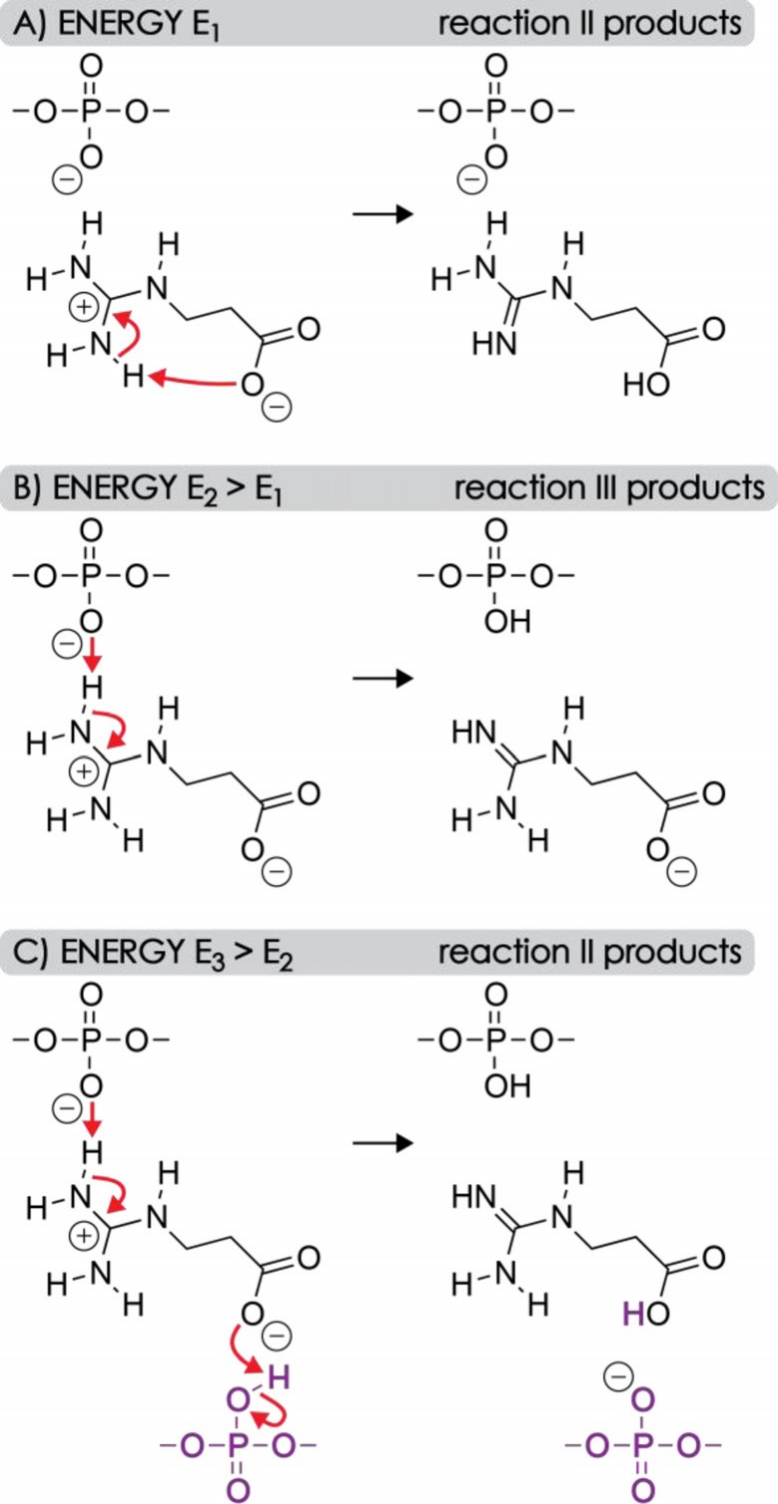
Proposed PT reactions associated with ligand dissociation from [RNA+Gpa−4 H]^4−^ ions with energy requirements of A) *E*_1_, B) *E*_2_, and C) *E*_3_, for which *E*_1_<*E*_2_<*E*_3_. At elevated energy *E*_3_, changes in the higher‐order RNA structure allow for PT from an RNA phosphodiester moiety (shown in violet) that was not initially bound to Gpa. Other RNA–ligand interactions that potentially stabilize the complex structures before and after PT are omitted for clarity.

**Scheme 3:**
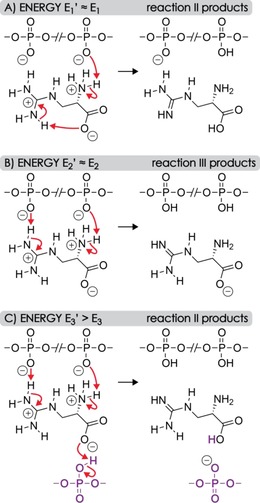
Proposed PT reactions associated with ligand dissociation from [RNA+aGpa−4 H]^4−^ ions with energy requirements of A) *E*_1_′, B) *E*_2_′, and C) *E*_3_′, for which *E*_1_′≈*E*_1_<*E*_2_′≈*E*_2_<*E*_3_<*E*_3_′. At elevated energy *E*_3_′, changes in the higher order RNA structure allow for PT from an RNA phosphodiester moiety (violet) that was not initially bound to aGpa. Other RNA–ligand interactions that potentially stabilize the complex structures before and after PT are omitted for clarity.

For Gpa, aGpa, and Arg at *n*=4, the fraction of [RNA+(*m*−1) L−(*n*−1) H]^(*n*−1)−^ and [RNA+(*m*−2) L−(*n*−2) H]^(*n*−2)−^ ions from Reaction (III) (of all products from Reactions (II) and (III)) generally increased with increasing energy used for CAD (Figure 2). For Gpa above ≈10 eV, however, the fraction of products from Reaction (III) substantially decreased again in favor of those from Reaction (II). At energies above 20 eV, ***c***, ***y***, ***a***, and ***w*** fragments from RNA backbone cleavage and loss of charged and neutral RNA nucleobases were observed (Figure 2), but these cannot account for the decrease in products from Reaction (III) because they were also observed in highly similar yields for aGpa and Arg. Moreover, the RNA ions from Reaction (II) have a higher net charge and thus are more prone to covalent‐bond cleavage than those from Reaction (III) (Figure S2), which should increase and not decrease the fraction of products from Reaction (III). Instead, we propose that the observed partitioning between products from Reactions (II) and (III) results from different energy requirements for the different PT reactions associated with ligand dissociation, as illustrated in Scheme 2 for Gpa and Scheme 3 for aGpa; reactions for Arg should be similar to those of aGpa.

As discussed above, Gpa, aGpa, and Arg have zwitterionic structures at the solution pH of ≈7.5 used here, and probably bind to deprotonated RNA by the formation of salt bridges. Based on the energies in Scheme 1, we propose that the PT reactions associated with ligand dissociation [Reactions (II) and (III)] occur in the gas phase. In Scheme 2A, the salt bridge between the guanidinium moiety of Gpa and a deprotonated RNA phosphodiester moiety is converted into a far weaker ionic hydrogen bond20b by an intramolecular PT from the guanidinium to the carboxylate moieties of Gpa, which dissociates into Reaction (II) products at relatively low energy (*E*_1_). At elevated energy (*E*_2_), an intermolecular PT between the guanidinium moiety of Gpa and the deprotonated RNA phosphodiester moiety becomes competitive and more products from Reaction (III) are observed (Scheme 2B). The proposed order of energies, *E*_2_>*E*_1_, is consistent with the higher PA of acetate (≈1454 kJ mol^−1^)^30^ compared with that of dimethylphosphate (1387 kJ mol^−1^),^39^ according to which PT from the guanidinium to the carboxylate moiety is energetically favored by 67 kJ mol^−1^ over PT to a deprotonated phosphodiester moiety. Although the PAs of small model compounds generally differ from those of the corresponding sites in [RNA+*m* L−*n* H]^*n*−^ ions, primarily as a result of hydrogen bonding and the presence of multiple charges,^36^ they can still reflect the competition for protons between different sites.

Importantly, the PT reactions in Scheme 2A and B do not require any changes in the RNA–ligand complex structure, whereas protonation of the carboxylate group in Scheme 2C assumes that an uncharged RNA phosphodiester moiety (or, alternatively, a nucleobase with relatively high gas‐phase acidity, such as guanine or adenosine)^40^ comes into sufficiently close proximity to the carboxylate during extension of the RNA structure^41^ at even higher energy (*E*_3_), which makes another proton available for intermolecular PT and Gpa dissociation by Reaction (II). The latter PT reaction was negligible for *m*=2 to 3, which we attribute to higher energy requirements for structural transitions in the [RNA+2 Gpa−4 H]^4−^ and [RNA+3 Gpa−4 H]^4−^ ions that are stabilized by additional electrostatic interactions. Likewise, CAD of [RNA+Gpa−5 H]^5−^ ions (Figure S3) showed only very few products from Reaction (II) at higher energy (Scheme 2C), which can be rationalized by the smaller number of protons in the [RNA−5 H]^5−^ ions compared with that of the [RNA−4 H]^4−^ ions, and an inherently more extended structure of the more highly charged nucleic acid anions.^42^

The proposed interactions and PT reactions associated with aGpa dissociation from [RNA+aGpa−4 H]^4−^ ions are illustrated in Scheme 3; those of Arg should be similar. In addition to the guanidinium moiety, both aGpa and Arg have an amino group that is protonated at pH 7.5^43^ and can form an additional salt bridge with another, not necessarily adjacent, deprotonated phosphodiester moiety. However, the PA of methylamine (899 kJ mol^−1^)^30^ as a model for the amino group is substantially smaller than that of methylguanidine (1002 kJ mol^−1^)^44^ as a model for the guanidinium moiety, and we propose that facile PT occurs at approximately the same energy as that required for intramolecular PT from the guanidinium to the phosphodiester moiety (*E*_1_′≈*E*_1_). At elevated energy (*E*_2_′≈*E*_2_), two protons are transferred to the RNA and [aGpa−H]^−^ dissociates. At energy *E*_3_′, which is significantly higher than *E*_3_ because extension of the RNA structure requires more energy when the additional amino group also forms a hydrogen bond with the RNA, a proton is transferred from a remote site and neutral aGpa dissociates.

Consistent with a higher stability of [RNA+*m* L−4 H]^4−^ ions of Gpa, aGpa, and Arg with *m*=2 and 3, very few products from Reaction (II) were observed at energies *E*_3_ and *E*_3_′ (Figure 2). Moreover, CAD of [RNA+*m* L−5 H]^5−^ ions of Gpa, aGpa, and Arg produced higher yields of [RNA+(*m*−1) L−(*n*−1) H]^(*n*−1)−^ and [RNA+(*m*−2) L−(*n*−2) H]^(*n*−2)−^ ions from Reaction (III) at all energies used (Figure S3), which can be rationalized by the smaller number of protons available for Reaction (II).

In summary, dissociation of [RNA+*m* L−*n* H]^*n*−^ ions of tmeGnd, meGnd, and Gnd at *n*=2 to 5 and all energies used gave only products from loss of neutral ligand L [Reaction (II)] by PT from [L+H]^+^ to a deprotonated phosphodiester moiety and subsequent dissociation of the [RNA+(*m*−1) L−*n* H]^*n*−^ **⋅**L complexes (Schemes 1 and 2B). Likewise, only products of Reaction (II) were observed for Gpa, aGpa, and Arg at *n*=2 to 3, but the PT reactions (Schemes 2A, C and 3A, C) involved in their formation include both intra‐ and intermolecular PT between aminium, guanidinium, carboxylate, and phosphodiester moieties. Finally, the competition between the latter reactions accounts for the energy‐dependent branching ratio between products from Reactions (II) and (III) in CAD of RNA complexes with Gpa, aGpa, and Arg at *n*=4 to 5.

### 2.3 Relative Stabilities of RNA–Ligand Complexes

**Figure 3:**
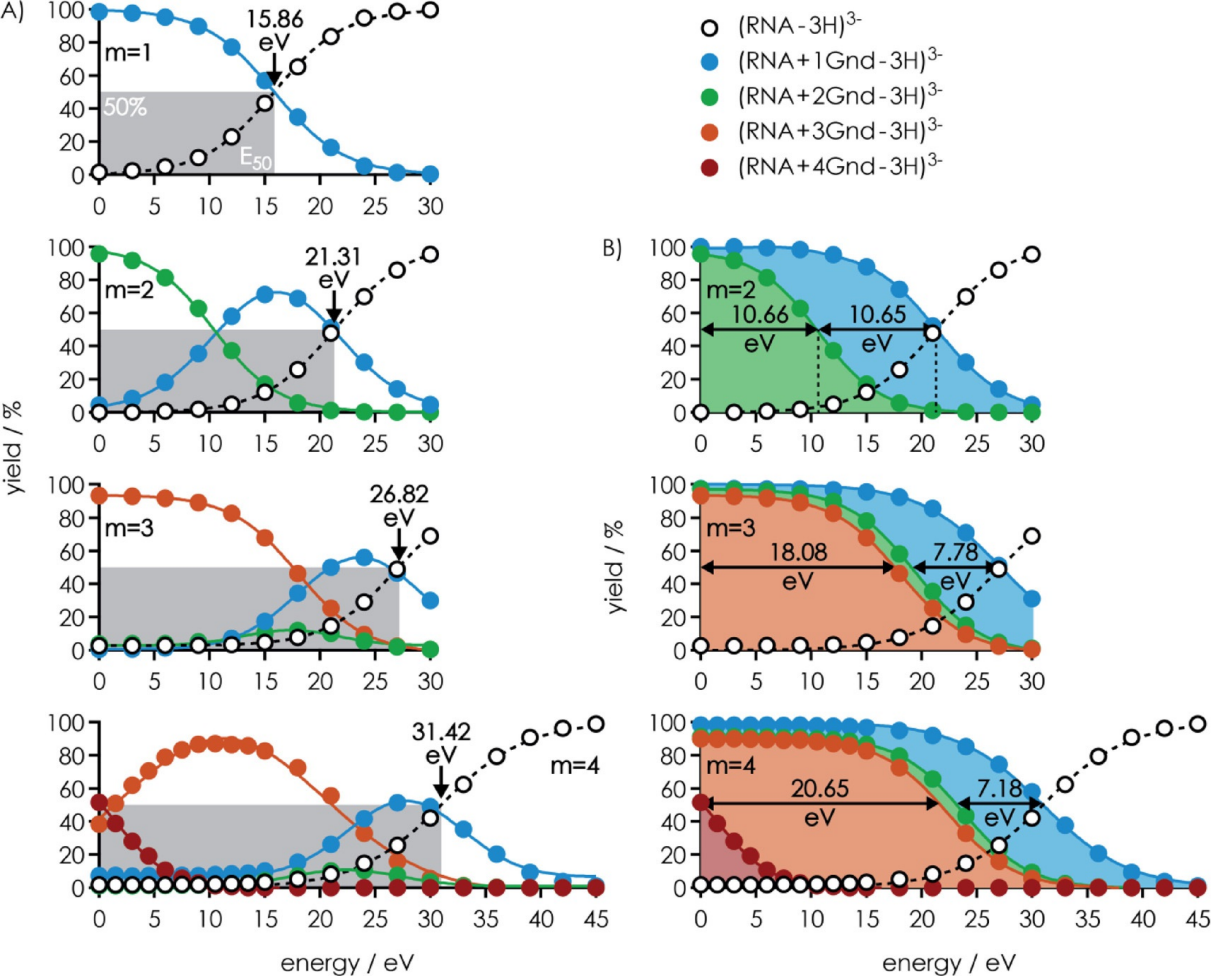
A) Yield of [RNA+*m* Gnd−3 H]^3−^ ions and its dissociation products from Reaction (II) at *m*=1–4, as indicated, versus laboratory‐frame energy used for CAD, and B) the same data with product yields plotted on top of each other.

As illustrated for [RNA+3 Gnd−3 H]^3−^ ions in Figure 1B, some unintended loss of ligand was observed after isolation of the RNA–ligand complex ions, which we attribute to vibrational excitation in the linear quadrupole used for ion isolation.^45^ The extent of ligand loss during isolation generally increased with an increase in the complex charge and number of ligands bound, and was always highest for tmeGnd (Table S2). However, in all experiments herein, the fraction of [RNA+*m* L−*n* H]^*n*−^ complex ions decreased sigmoidally with increasing energy used for CAD (Figure 3A); similar breakdown curves have been observed in CAD of noncovalent complexes^46^ comprised of DNA and basic amino acids or small peptides,^47^ phosphopeptides and basic ligands,17a duplex DNA and minor groove binders,^48^ and RNA^49^ and DNA^50^ duplexes.

Because [RNA−3 H]^3−^ ions were the only products from CAD of [RNA+1 Gnd−3 H]^3−^ ions in the energy range investigated, the sigmoidal breakdown curve for [RNA+1 Gnd−3 H]^3−^ ions is exactly the inverse of the appearance curve for [RNA−3 H]^3−^ ions, with a common *E*_50_ value of ≈15.86 eV. As illustrated in Figure 3A, these data can be fitted with a sigmoidal function without vertical offset and a decay rate *r*, that is, Equation (1):

(1) $$Y\left(x\right)=100\;(1+\exp\left(\left(E{}_{50}-x\right)/r\right){}^{-1}$$

In this case, the sigmoidal function is 0 % (and the inverse 100 %) at low energy and plateaus at 100 % (inverse 0 %) at high energy, and *E*_50_ is the energy value at 50 % yield. However, Equation (1) does not account for the unintended loss of ligand discussed above; for example, in CAD of [RNA+4 Gnd−3 H]^3−^ ions, the yield of free RNA ions, [RNA−3 H]^3−^, was constantly 1.816 % (±0.005 % standard deviation) at energies of up to 10 eV (Figure 3A). Fitting these data with the function in Equation (2):

(2) $$Y\left(x\right)=a+\left(100-a\right)(1+\exp\left(\left(E{}_{50}-x\right)/r\right){}^{-1}$$

accounts for the vertical offset of the sigmoid, and indicates an *E*_50_ value at the inflection point of the sigmoid of (31.42±0.05) eV instead of the energy value at 50 % yield of (30.98±0.13) eV from fitting the data with Equation (1). Thus the CAD data were fit by using Equation (2) unless the number of data points in the plateau region was too small to gauge potential offsets.

CAD of [RNA+2 Gnd−3 H]^3−^ ions produced mostly [RNA+1 Gnd−3 H]^3−^ ions at energies of up to 15 eV, but above this energy, [RNA−3 H]^3−^ ions became more abundant, consistent with sequential dissociation of Gnd ligands [Reaction (II)]. To determine the relative energies required for the dissociation of each Gnd ligand, the data were plotted by sequentially adding the yields of all species except that of free RNA. In this representation (Figure 3B), the sequentially added yields were best described by sigmoidal functions of the type shown in Equation (3):

(3) $$Y\left(x\right)=\left(100-b\right)(1+\exp\left(\left(E{}_{50}-x\right)/r\right){}^{-1}$$

For CAD of [RNA+2 Gnd−3 H]^3−^ ions, the *E*_50_ values from this analysis revealed that within error limits, the energies required for dissociation of the first and the second Gnd are the same, approximately 10.65 eV. By contrast, the energy requirements for sequential dissociation of Gnd from [RNA+*m* Gnd−3 H]^3−^ ions with *m*=3 and 4 are vastly different (Figure 3B). For *m*=3, the first and second Gnd ligands dissociated at nearly the same energy (*E*_50_ values of (18.08±0.06) and (19.40±0.07) eV), whereas for *m*=4, the first Gnd ligand dissociated at a far lower energy of (1.75±0.26) eV, and the second and third Gnd ligands dissociated at very similar energies, (22.40±0.04) and (23.80±0.03) eV, respectively. Moreover, there is no apparent systematic trend in the *E*_50_ values for the breakdown of [RNA+*m* Gnd−3 H]^3−^ ions with *m*=1 to 4, that is, (15.86±0.08), (10.66±0.06), (18.08±0.06), and (1.75±0.26) eV for *m*=1, 2, 3, and 4, respectively (Figure 3A).

**Figure 4:**
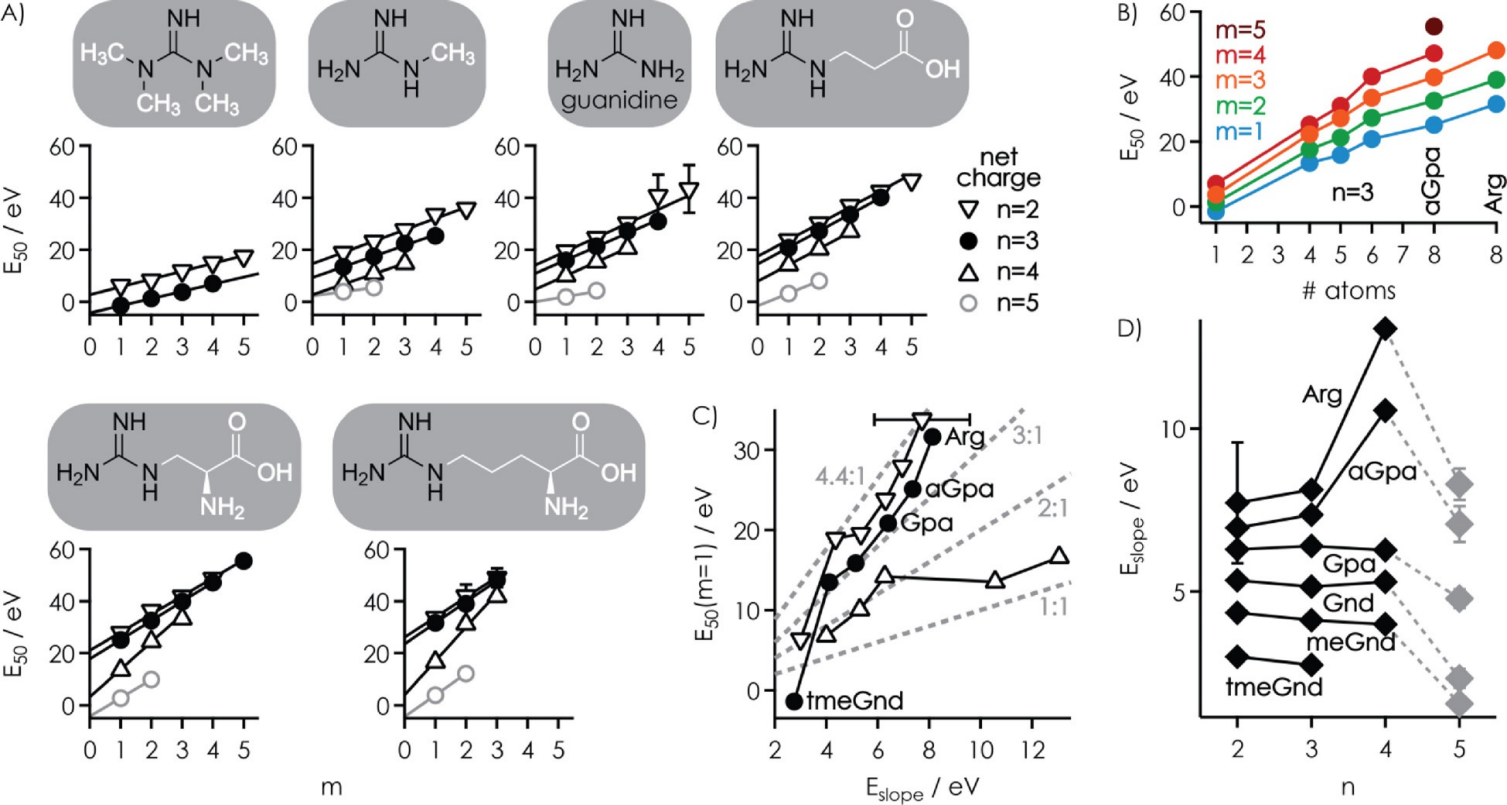
A) *E*_50_ values for the dissociation of all ligands by CAD of [RNA+*m* L−*n* H]^*n*−^ complex ions with *n*=2–5 versus the total number of ligands *m* (corresponding ligand structures with the guanidine moieties highlighted in black on top), B) *E*_50_ values for *n*=3 versus the number of atoms that can potentially be involved in hydrogen‐bond or salt‐bridge interactions with the RNA for *m*=1–5, C) *E*_50_ values at *m*=1 versus slope values *E*_slope_ from linear‐fit functions in (A) for *n*=2–4; the dashed lines indicate *E*_50_(*m*=1)/*E*_slope_ 1:1, 2:1, 3:1, and 4.4:1, D) *E*_slope_ versus complex net charge *n* (values at *n*=5 are shown in gray to highlight a systematic decrease in the values of *E*_slope_).

However, the *E*_50_ values for the appearance of [RNA−3 H]^3−^ ions, which correspond to the energies required to dissociate all Gnd ligands bound to the RNA, increased linearly with increasing *m* (Figure 4A). This means that irrespective of the complexity of the reaction coordinates for [RNA+*m* Gnd−3 H]^3−^ ion dissociation, each additional Gnd ligand increased the energy required for dissociation by a fixed amount (*E*_slope_) that was, within error limits, independent of the total number of ligands initially bound to the RNA except for *m*=1. Similar behavior was observed for all other ligands and complex net charges studied, although the *E*_50_ values generally increased in the order tmeGnd<meGnd<Gnd<Gpa<aGpa<Arg (Figure 4A). Importantly, this order of complex stability is inconsistent with the order of PA (1032, 986, and 1051 kJ mol^−1^ for tmeGnd, Gnd, and Arg, respectively),^28^ but instead shows an almost linear correlation with the number of ligand atoms that can potentially be involved in hydrogen‐bond or salt‐bridge interactions with the RNA (tmeGnd: 1; meGnd: 4; Gnd: 5; Gpa: 6; aGpa: 8; Arg: 8), as illustrated in Figure 4B for *n*=3 (Figure S4 shows data for *n*=2 and 4). The higher stability of Arg versus aGpa [RNA+*m* L−*n* H]^*n*−^ complexes can be attributed to the longer alkyl chain of Arg that allows it to better adapt to the RNA structure^51^ and reach more binding sites.^52^ This correlation does not exclude the presence of stacking, cation–π, or other noncovalent interactions, but suggests that hydrogen bonds and salt bridges provide the largest contribution to complex stability.

Because the *E*_50_ values are a relative measure of the minimum energy required for complex dissociation, we conclude that the contribution of [RNA−*n* H]^*n*−^ **⋅**L binding energy to complex stability is significantly higher than that of Δ*H*_PT,complex_ (Scheme 1), and that the differences in the binding energy of the different ligands primarily result from differences in the number of hydrogen‐bond and salt‐bridge interactions that they can form. Moreover, for each ligand, the *E*_50_ values systematically decreased as net charge *n* increased (Figure 4A), which is consistent with an increasing PA of [RNA−*n* H]^*n*−^ ions with increasing *n* (as also indicated by their PT reactivity in the collision cell, discussed above) that in turn decreases the binding energy of [RNA−*n* H]^*n*−^ **⋅**L complexes (Scheme 1).

Although each additional ligand increased the energy required for dissociation of all ligands by a fixed *E*_slope_ value (within error limits), the linear‐fit functions in Figure 4A did not generally extrapolate to 0 eV at *m*=0 but showed intercept energies as small as (−4.23±0.09) eV and as large as (26.03±2.82) eV. In other words, one of the *m* ligands (including that for *m*=1) can bind to the RNA more strongly than all others, that is, when *E*_50_(*m*=1)>*E*_slope_ or, for tmeGnd at *n*=3 for which *E*_50_(*m*=1)<*E*_slope_, more weakly than all others (Figure 4C). This strongly suggests that the 8‐nt RNA provides a single, unique binding site to which only one of the *m* ligands binds preferentially, along with four other binding sites to which up to four ligands can bind. With the exception of tmeGnd at *n*=3, binding to the unique site was always stronger than binding to the other four sites by a factor of up to ≈4.4 (Figure 4C).

**Scheme 4:**
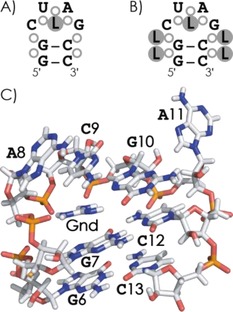
Possible RNA hairpin structure with A) one ligand bound to the phosphodiester/ribose moieties (shown as open circles) in the loop region and B) four additional ligands bound to the phosphodiester/ribose moieties of the stem. C) Truncated structure of the *Gloeobacter violaceus* guanidine II riboswitch P1 stem–loop with guanidine^55^ (pdb structure 5NEF).

A possible RNA structure that agrees with all the experimental data from this study is the hairpin motif illustrated in Scheme 4, with a stem that consists of only two G‐C base pairs and a CUAG loop to provide a unique binding site. The CUAG loop has the potential for hydrogen‐bonding interactions similar to those of the highly stable UUCG loop, and 12‐nt hairpin structures with the former (GGAC‐CUAG‐GUCC, melting temperature *T*_m_=(69.8±1.0) °C) are only slightly less stable than hairpin structures with the latter (GGAC‐UUCG‐GUCC, *T*_m_=(72.9±1.0) °C).^53^ Hairpin structures with UUCG loops have a minimum requirement of a stem comprised of two base pairs, with melting temperatures of ≈24, ≈54, and ≈55 °C for CG‐UUCG‐CG, CC‐UUCG‐GG, and GC‐UUCG‐GC, respectively.^54^ Although theory predicts no stable secondary structure for our GGCUAGCC RNA by itself, a hairpin fold could nevertheless be stabilized by binding of guanidinium ligands. For example, the crystal structure of a hairpin motif for guanidine binding, ‐GG‐ACGA‐CC‐, in which guanidine interacts with all three phosphodiester moieties of the ACGA loop and is stacked upon the guanine base on the 5′ side of the loop in a cation–π interaction (Scheme 4C), has been reported for an 18‐nt guanidine‐II riboswitch.^55^

A similar hairpin structure for our 8‐nt RNA (GG‐CUAG‐CC), in which the guanidine moiety of the ligands at *m*=1 can interact with all three phosphodiester moieties of the loop (Scheme 4A), explains the unique binding site, whereas the exposed phosphodiester moieties of the stem can account for the binding of up to four additional ligands (Scheme 4B). Specifically, the binding pattern in Scheme 4B is consistent with similar binding strengths for the additional ligands that give rise to the *E*_slope_ values, and the approximately threefold stronger binding of the ligands at *m*=1 (Figure 4C). Finally, a hairpin structure agrees with the weaker binding of tmeGnd at *m*=1 because tmeGnd cannot form more than one salt‐bridge interaction with the phosphodiester moieties of the loop, and with the binding of up to only three instead of five Arg ligands (Figure 4A) because each additional Arg can bind to two adjacent phosphodiester moieties of the stem (Scheme 3). Any differences in the *E*_50_ values between different ligands can then be attributed to different numbers and strengths of interactions with the phosphodiester moieties, and to additional interactions with adjacent ribose moieties.

The slopes of the linear fit functions in Figure 4A were largely independent of RNA complex ion net charge for meGnd, Gnd, and Gpa at *n*=2 to 4, and for tmeGnd, aGpa, and Arg at *n*=2 to 3 (Figure 4D). In these complexes, the stabilization achieved by hydrogen‐bond and salt‐bridge interactions apparently dominates over any effects of the complex ion net charge for *m*>1 but not *m*=1 (Figure 4C), which suggests that specific binding (*m*=1) is far more affected by complex net charge than the binding of additional ligands (*m*>1). This is again consistent with the hairpin structures shown in Scheme 4, in which the charge density around the specifically bound ligand is much higher than that around the ligands bound to the phosphodiester moieties of the stem region. By contrast, at *n*=5, all slope values were significantly smaller than those at *n*=4, which indicated that Coulombic repulsion limits overall complex ion stability at *n*=5. Moreover, both the *E*_50_(*m*=1) and *E*_slope_ values for aGpa and Arg at *n*=4 stand out, whereas those at *n*=2 and 3 follow the general trends discussed above (Figure 4C, D). The *E*_50_(*m*=1) and *E*_slope_ ratio of close to one at *n*=4 is inconsistent with a unique binding site and instead suggests binding of up to three aGpa or Arg ligands to a largely extended RNA structure.

### 2.4 Sequential Dissociation of Ligands

As discussed by Rodgers and Armentrout^56^ and Kitova and Klassen,^57^ the potential energy surface for noncovalent bond cleavage has a staircase appearance; that is, there should be no reverse activation barriers and endothermic noncovalent complex dissociation generally proceeds once the available energy exceeds the thermodynamic threshold. In the above CAD experiments, in which all ligands were dissociated (Figure 4), we thus probed thermodynamic complex stability even though the energy values obtained from the *E*_50_ analysis are relative rather than absolute.^46^

**Figure 5:**
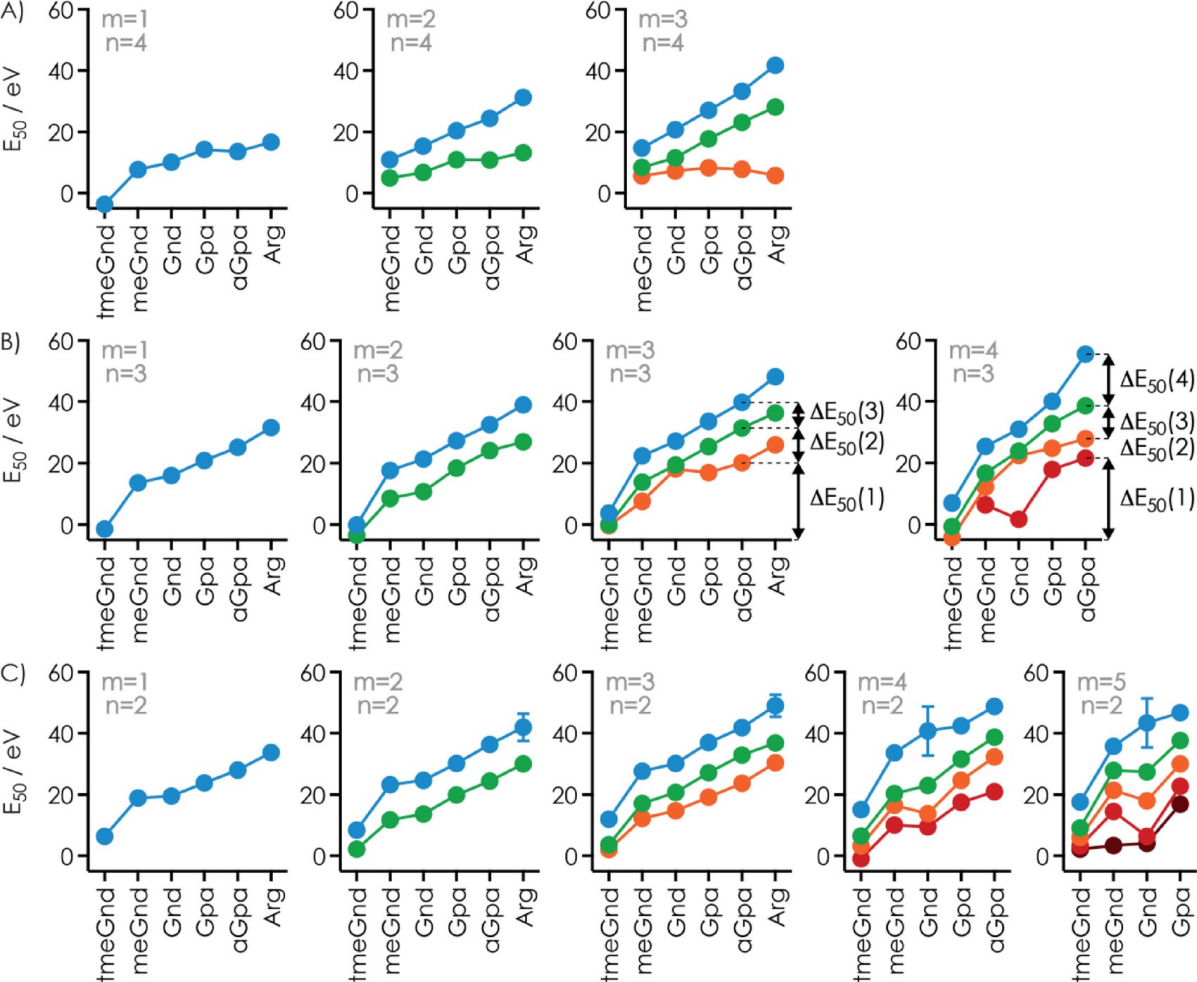
A) *E*_50_ values for the sequential dissociation of individual ligands (blue: ligand *m*, green: ligand *m*−1, orange: ligand *m*−2, red: ligand *m*‐3, brown: ligand *m*−4) in CAD of [RNA+*m* L−*n* H]^*n*−^ complex ions with *m*=1–5 and A) *n*=4, B) *n*=3, and C) *n*=2; Δ*E*_50_ values for the sequential dissociation of individual aGpa ligands at *n*=3 and *m*=3 and 4 are indicated by arrows.

However, the *E*_50_ values for sequential dissociation of individual ligands, summarized for tmeGnd, meGnd, Gnd, Gpa, aGpa, and Arg at *n*=2 to 4 and *m*=1 to 5 in Figure 5, do not generally indicate relative binding energies of individual ligands. This is evident from the strong effect of the initial number of ligands, *m*, on the Δ*E*_50_ values (Figure 5). For example, for aGpa at *n*=3 and *m*=3, dissociation of the first ligand was observed at an Δ*E*_50_(1) value of (20.09±0.16) eV, dissociation of the second ligand was observed at an additional Δ*E*_50_(2) value of (11.37±0.26) eV, and dissociation of the third ligand was observed at an additional Δ*E*_50_(3) value of (8.40±0.36) eV. By contrast, the values of Δ*E*_50_(1), Δ*E*_50_(2), and Δ*E*_50_(3) for aGpa at *n*=3 and *m*=4 were (20.51±0.23), (6.40±0.46), and (10.76±0.85) eV, respectively (Figure 5B). Strikingly, the Δ*E*_50_(2) value at *m*=4 (6.40 eV) was only 56 % of the Δ*E*_50_(2) value at *m*=3 (11.37 eV), whereas the Δ*E*_50_(3) value at *m*=4 (10.76 eV) was larger by 28 % than the Δ*E*_50_(3) value at *m*=3 (8.40 eV).

However, by far the most irregular energy differences for Δ*E*_50_ were found for Gnd at *m*≥3 (Figure 3B, Figure 5), and for aGpa and Arg at *n*=4 (Figure 5). A possible rationale for this observation are intricate conformational rearrangements of the RNA during sequential [RNA+*m* L−*n* H]^*n*−^ ion dissociation along with ligand scrambling and PT reactions not only between ligands and the RNA (Schemes 2C and 3C) but also between ligands. As a case in point, the multidentate [Gnd+H]^+^ ion should be especially prone to scrambling and PT between Gnd ligands because of its high symmetry. Such structural rearrangements would allow for the dissociation of individual ligands at energies that can be higher or lower than the average energy required for ligand dissociation, without changing the total energy required for dissociation of all ligands.

### 2.5 RNA Backbone Cleavage at Elevated Energy

**Figure 6:**
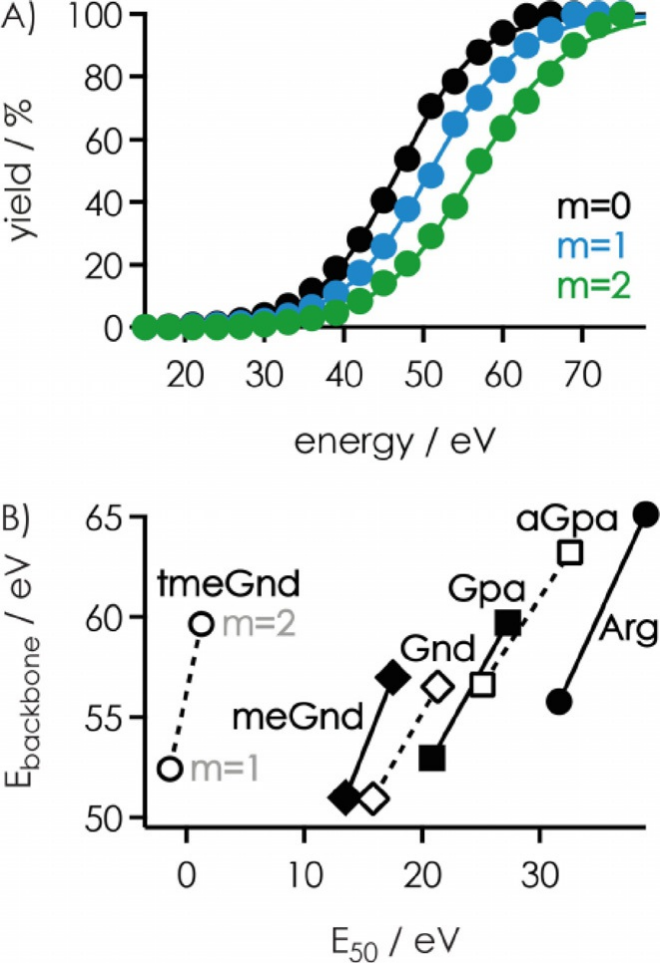
A) Yields of ***c***, ***y***, ***a***, and ***w*** fragments from backbone cleavage, including those that showed nucleobase loss, from CAD of [RNA−3 H]^3−^ (black), [RNA+1 Gnd−3 H]^3−^ (blue), and [RNA+2 Gnd−3 H]^3−^ (green) ions versus laboratory‐frame energy, B) *E*_50_ values for the appearance of fragments from backbone cleavage (*E*_backbone_) versus *E*_50_ values for the dissociation of all ligands in CAD of [RNA+*m* L−3 H]^3−^ ions with *m*=1 and 2.

CAD of [RNA+*m* L−*n* H]^*n*−^ ions also produced ***c***, ***y***, ***a***, and ***w*** fragments from RNA backbone cleavage and loss of charged and neutral RNA nucleobases at elevated energy (Figure 2). For [RNA+*m* L−3 H]^3−^ ions with *m*=0 to 2, we determined *E*_50_ values for the appearance of fragments from RNA backbone cleavage (Figure 6A). These *E*_backbone_ values were significantly higher, by a factor of 1.7 (Arg) to 46.5 (tmeGnd), than those for the dissociation of all ligands (Figure 6B), from which we conclude that ligand dissociation and backbone cleavage are sequential processes. Surprisingly, the *E*_backbone_ values increased in the order Gnd<tmeGnd<Arg, which is also the order of PA (986, 1032, and 1051 kJ mol^−1^ for Gnd, tmeGnd, and Arg, respectively). This indicates that PT to the RNA (Schemes 2 and 3) does affect backbone cleavage, most likely by facilitating nucleophilic attack of 2′‐OH groups on adjacent phosphorus atoms.^41^ Specifically, the timing between PT (Schemes 2 and 3) and nucleophilic attack should depend on the value of Δ*H*_PT,complex_ and thus ligand PA; facilitation of nucleophilic attack can only occur if the proton is transferred before nucleophilic attack.

## 3 Conclusions

Our comprehensive study shows that ESI and CAD can be used to obtain detailed information on RNA–ligand binding. For tmeGnd, meGnd, Gnd, Gpa, aGpa, and Arg ligands in mixtures with an 8‐nt RNA, the ESI data suggest that the gaseous [RNA+*m* L−*n* H]^*n*−^ complex ions predominantly originate from association reactions in solution by the formation of intermolecular salt bridges between the ligand guanidinium and RNA phosphodiester moieties. The order of [RNA+*m* L−*n* H]^*n*−^ complex stability, tmeGnd<meGnd<Gnd<Gpa<aGpa<Arg, established in the CAD experiments, revealed that salt bridges and hydrogen bonds provide the largest contribution to complex stability in the gas phase, whereas ligand PA showed some effect only on RNA backbone cleavage at elevated energy. Ligand dissociation in CAD of [RNA+*m* L−*n* H]^*n*−^ complex ions was generally accompanied by PT from ligand to RNA, for which we have proposed mechanisms that also account for the energy‐dependent competition between neutral versus deprotonated ligand loss of Gpa, aGpa, and Arg at *n*=4. Evidence for ligand scrambling during CAD, particularly for the highly symmetric Gnd, was also found, although scrambling did not change the total energy required for ligand dissociation. Moreover, data from CAD of [RNA+*m* L−*n* H]^*n*−^ complex ions with *m*=1 to 5 indicate an RNA structure to which one of the *m* ligands binds more strongly than all others; a hairpin motif is consistent with this observation. In future experiments, we plan to study ligands of increased molecular complexity, such as diarginine, together with different RNA sequences to gain further insight into RNA–ligand binding and complex stability in the gas phase.

## Experimental Section

Experiments were performed on a 7 T Fourier transform ion cyclotron resonance (FT‐ICR) mass spectrometer (Bruker, Austria) equipped with an ESI source for [M−*n* H]^*n*−^ ion generation, a linear quadrupole for ion isolation by *m*/*z*, and a collision cell floated with Ar gas for CAD. RNA–ligand complexes were electrosprayed at a flow rate of 1.5 μL min^−1^ from solutions of RNA (1 μm) and ligand (5–100 μm) in 1:1 CH_3_OH/H_2_O at pH ≈7.5 adjusted by the addition of ≈1 mm piperidine and imidazole each. Methanol was HPLC grade (Acros, Vienna, Austria), H_2_O was purified to 18 MΩ cm^−1^ at RT by using a Milli‐Q system (Millipore, Austria), and all ligand compounds (1,1,3,3‐tetramethylguanidine, >99 %; 1‐methylguanidine hydrochloride, 98 %, guanidine hydrochloride, ≥99 %; 3‐guanidinopropionic acid; ≥99.5 %; l‐2‐amino‐3‐guanidinopropionic acid hydrochloride; ≥99.5 %; l‐arginine monohydrochloride, ≥99 %) were purchased from Sigma–Aldrich (Vienna, Austria). The 8‐nt RNA (5′‐GGCUAGCC‐3′ with HO termini) was prepared by solid‐phase synthesis using 2′‐*O*‐[(triisopropylsilyl)oxy]methyl (TOM) chemistry, purified by HPLC, and desalted by using Sep‐Pak C18 cartridges, washed with triethylammonium bicarbonate (0.1–0.15 m) in H_2_O, and eluted with 1:1 CH_3_CN/H_2_O.^58^ Between 50 and 100 scans were added for each spectrum, and data reduction used the SNAP2 algorithm (Bruker, Austria). Errors are standard deviations from linear (*E*_slope_) or nonlinear (*E*_50_) least‐squares fitting procedures.

## Conflict of interest

*The authors declare no conflict of interest*.

## Supporting information

As a service to our authors and readers, this journal provides supporting information supplied by the authors. Such materials are peer reviewed and may be re‐organized for online delivery, but are not copy‐edited or typeset. Technical support issues arising from supporting information (other than missing files) should be addressed to the authors.

Supplementary
