# Supplementary material for: Interactions of Protonated Guanidine and Guanidine Derivatives with Multiply Deprotonated RNA Probed by Electrospray Ionization and Collisionally Activated Dissociation
Source: ChemistryOpen. 2017 Oct 24;6(6):739–50. doi: 10.1002/open.201700143 (PMC5715244; doi:10.1002/open.201700143)
Supplement: Supplementary file 1 — Supplementary [file OPEN-6-739-s001.pdf]

ChemistryOPEN

Supporting Information

© 2017 The Authors. Published by Wiley-VCH Verlag GmbH & Co. KGaA, Weinheim

Interactions of Protonated Guanidine and Guanidine Derivatives with Multiply Deprotonated RNA Probed by Electrospray Ionization and Collisionally Activated Dissociation

Jovana Vušurović, Eva-Maria Schneeberger, and Kathrin Breuker*[a]

open_201700143_sm_miscellaneous_information.pdf

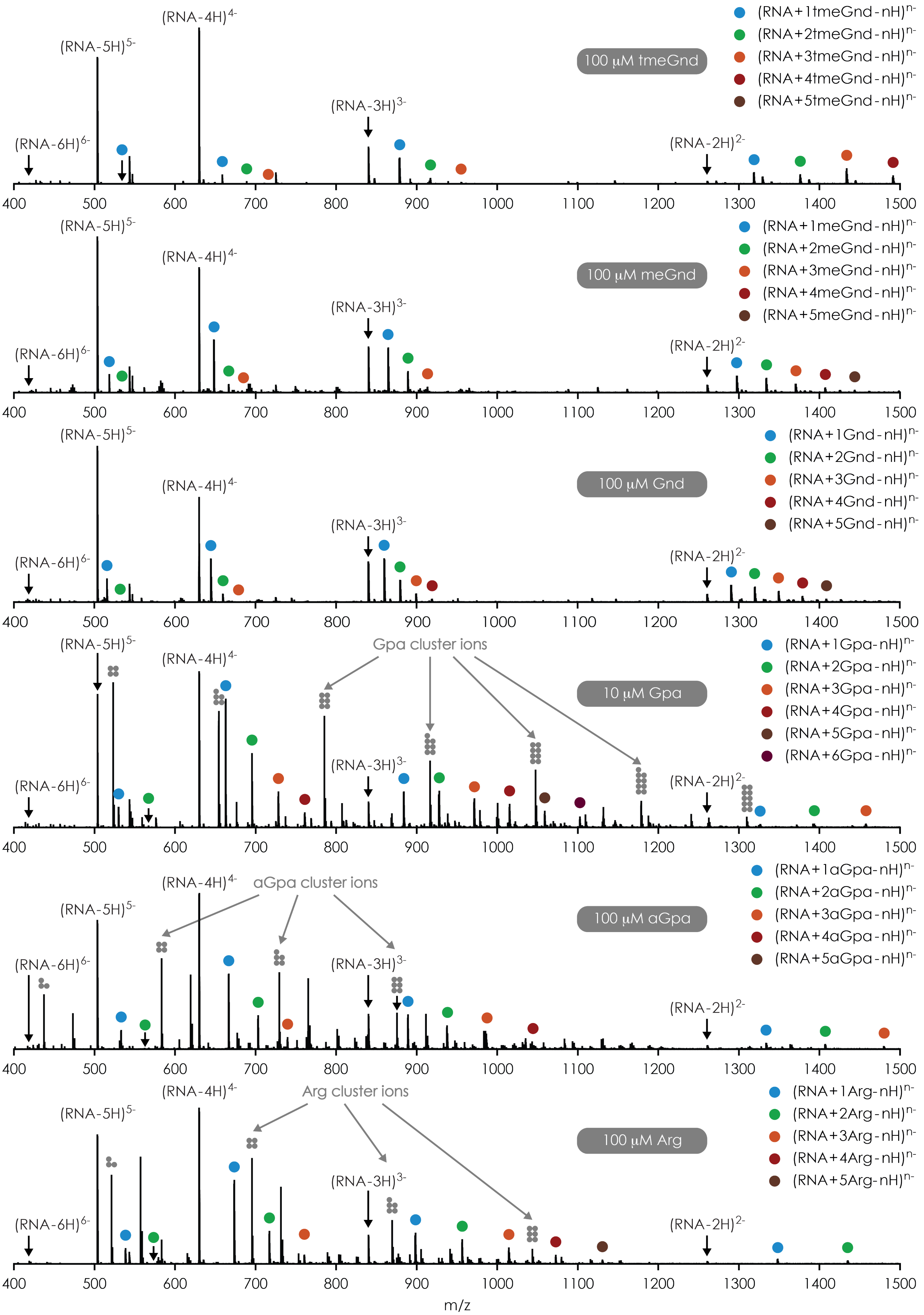

Figure S1. Mass spectra from electrospray ionization of 1 µM RNA solutions in 1:1 H_2O/CH_3OH with 100 µM tetramethylguanidine (tmeGnd), 1-methylguanidine (meGnd), guanidine (Gnd), 1,1,3,3-L-2-amino-3-guanidinopropanoic acid (aGpa), L-arginine (Arg), and 10 µM 3-guanidinopropanoic acid (Gpa) at pH ~7.5; colored circles indicate the number of ligands bound to the 8 nt RNA and grey circles indicate singly charged ligand cluster ions.

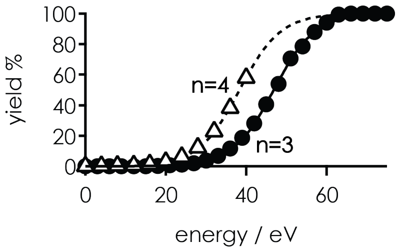

Figure S2. Yield of ***c***, ***y***, ***a***, and ***w*** fragments from RNA backbone cleavage and loss of charged and neutral RNA nucleobases from CAD of (RNA-nH)$^{n-}$ ions at n=3 and 4 versus energy, illustrating the higher stability of (RNA-3H)$^{3-}$ over (RNA-4H)$^{4-}$ ions, the CAD data at n=3 are the same as in Figure 6A for m=0.

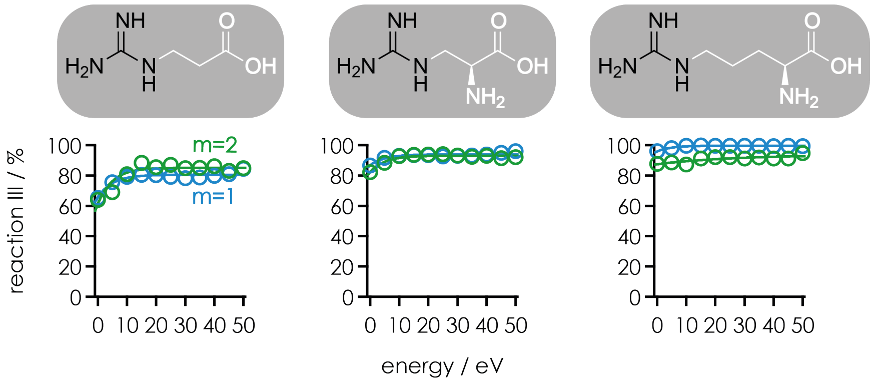

Figure S3. Fraction of products from reaction III out of all products from reactions II and III (left axes) in CAD of (RNA+mL-5H)$^{5-}$ ions for m=1 (blue) and m=2 (green) for Gpa, aGpa, and Arg versus laboratory frame energy.

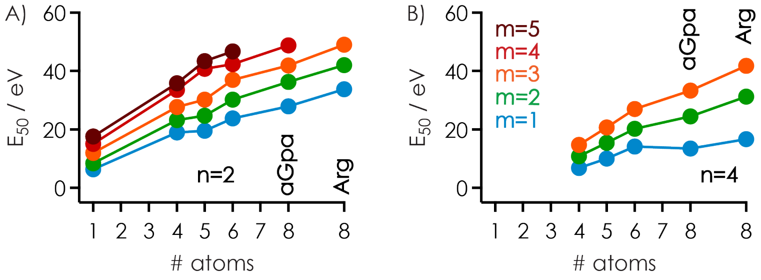

Figure S4. E_{50} values for dissociation of all ligands in CAD of (RNA+mL-nH)$^{n-}$ complex ions with m=1-5 for A) n=2 and B) n=4 versus the number of atoms that can potentially be involved in hydrogen bond or salt bridge interactions with the RNA.

Table S1. From ESI of 1 μM RNA and 5-100 μM ligand solutions in 1:1 H_2O/CH_3OH at pH 7.5, fraction of (RNA+mL-nH)$^{n-}$ ions with m≥1 for each n.

ligand	ligand concentration / μM	n=2	n=3	n=4	n=5	n=6
tmeGnd	100	94.3	48.8	6.2	0.1	0
meGnd	100	86.4	62.2	33.7	11.4	0
Gnd	100	87.0	66.7	33.7	13.8	0
Gpa	5	78.9	52.2	30.4	6.8	0
	10	94.1	88.0	63.7	14.8	0
aGpa	50	77.6	68.1	39.2	9.4	0
	100	81.6	73.9	45.2	14.2	0
Arg	50	73.6	65.6	40.1	9.6	0
	100	81.3	76.2	46.1	11.5	0

Table S2. Extent of unintended ligand loss during isolation in the linear quadrupole.

Complex charge state (n)	Number of ligands (m)	tmeGnd	meGnd	Gnd	Gpa	aGpa	Arg
2	5	78.76	32.95	89.09	0	-	-
	4	63.90	16.62	10.61	0	28.24	-
	3	48.25	9.61	0	0	24.18	16.19
	2	48.75	3.38	0	0	11.39	17.33
	1	12.71	0	0	0	4.63	12.38
3	4	99.43	53.22	48.37	11.48	16.43	-
	3	92.69	26.55	6.75	13.46	10.63	7.36
	2	86.07	19.17	4.35	9.48	11.56	10.89
	1	65.02	4.29	1.62	1.03	3.85	7.64
4	3	99.66	86.86	56.32	27.42	18.13	22.00
	2	98.92	44.48	21.60	12.91	11.41	7.65
	1	88.17	18.27	6.53	4.51	6.31	5.22
5	2	100	88.30	75.87	91.16	90.28	82.90
	1	100	50.90	39.00	68.70	65.63	29.35
